# A retrospective study on transabdominal ultrasound measurements of the rumen wall thickness to evaluate chronic rumen acidosis in beef cattle

**DOI:** 10.1186/s12917-020-02561-7

**Published:** 2020-09-15

**Authors:** Enrico Fiore, Vanessa Faillace, Massimo Morgante, Leonardo Armato, Matteo Gianesella

**Affiliations:** 1 Department of Animal Medicine, Productions and Health (MAPS), University of Padua, Viale dell’Università 16, 35020 Legnaro (PD), Italy; 2 Veterinary Freelance, Viale dell’Università 16, 35020 Legnaro (PD), Italy

**Keywords:** Beef cattle, Chronic rumen acidosis, Rumen wall, Ultrasonography

## Abstract

**Background:**

Chronic and subacute rumen acidosis are economically important in the beef industry. The aim of this study was to evaluate the potential suitability of the transabdominal ultrasonographic examination of the ruminal wall to diagnose chronic rumen acidosis in beef cattle compared to direct measurement of ruminal pH, as a fast non-invasive tool to be used in field condition. Ultrasonographic examination of the rumen was conducted in 478 beef cattle before rumenocentesis (chronic rumen acidosis group = pH ≤ 5.8; healthy group = pH ≥ 5.9). Rumen wall ultrasound measurements included rumen wall thickness (RWT) and rumen mucosa and submucosa thickness (RMST).

**Results:**

The Analysis of Variance showed the high significant effect of the pH class for RWT and RMST (*P* < 0.001). Spearman RANK correlation analysis showed interaction between rumen pH and RWT (− 0.71; *P* < 0.0001) and RMST (− 0.75; P < 0.0001). A significant Spearman’s correlations were found between volatile fatty acids (VFA) and RWT and RMST.

The differentiation efficiency of RWT between healthy and chronic rumen acidosis groups, as a result of the receiver operator curve (ROC) analysis, was quite good with an area under the receiver operator curve (AUROC) of 0.88: *P* < 0.0001; 95% CI: 0.83–0.98. Using a cut-off value of > 8.2 mm. The differentiation efficiency of RMST between healthy and chronic rumen acidosis groups, as a result of ROC curve analysis, was good with an AUROC of 0.90: *p* < 0.0001; 95% CI: 0.85–0.94. Using a cut-off value of > 5.3 mm.

**Conclusions:**

In this study, the thickening of RWT and RMST is correlated with the changes of ruminal pH. Transabdominal rumen ultrasound has the potential to become a powerful diagnostic tool useful to identify fattening bulls affected by chronic rumen acidosis.

## Background

In the livestock breeding of beef cattle, the intensive production system is commonly used in Italy. This farming method includes often the importation of beef cattle from northern Europe at the age of 8–15 months and taken to the finishing stage and slaughter [1]. A critical phase is the restocking period after transportation and the first 20 to 30 days following housing [1, 2]. The growth of animals is subject to many stressors (different conditions of temperature, humidity, environment, transport, the change from an acclimation diet rich in fiber to a growth diet rich in concentrate, social interactions, and diseases), which change the physiological homeostasis and compromise the health status in restocking phase [2].

High-concentrate diets can cause metabolic disorders such as rumen acidosis [3] and bloat [4]. Ruminal acidosis is the most common condition caused precisely by high intake of grains rich in starch and low consumption of physically effective fiber [3]. These factors result in increased yields of VFA and lactic acid in the rumen. In addition, the low diet in long fiber decreases the time in the chewing and the relative saliva production is lower than normal. The negative effect on the rumen pH is because of the inadequate saliva production needed to neutralize the acids [5, 6]. High-concentrate diets can lead to increased rumen papillae length and width in response to changes in VFA concentration and ruminal fluid pH [7].

The ruminal acidosis could be defined as a digestive tract disorder that negatively affects ruminal fermentation, animal health, production and corporate profit [8]. Acidosis of ruminants often is separated into several forms, including acute, chronic or subacute ruminal acidosis [9]. The diagnosis of subacute ruminal acidosis is difficult under farm conditions as clinical signs are commonly subtle and delayed after the time of the acidotic insult [10].

Ruminal pH is the most commonly used parameter for detecting, and it can be analyzed with different methods. The most useful cut-off point between normal and abnormal has been defined as pH ≤5.5 [11]. A ruminal pH < 5.8 is referred to as indicating a critical pH-situation on a farm and it suggests a serious situation developing problem of subacute rumen acidosis [5, 9].

There are two principal methods to collect fluid samples: by an oral tube or by percutaneous rumenocentesis. Rumenocentesis is considered the golden test to perform the diagnosis of subacute rumen acidosis [5, 12] as it avoids contamination of the ruminal fluid by saliva (basic pH) which is ingested when the oral probe passes through the oral cavity and esophagus [13]. More studies considered rumenocentesis as an invasive and not-routine procedure with severe complications [14]. Although other recent studies have used this technique without significant welfare concerns or adverse effects on health and production in dairy cattle [15, 16], it is not yet used and implemented in beef cattle [1].

In recent years, ultrasonography has applied widely available in daily food animal practice. It is an ideal diagnostic tool for the investigation of bovine gastrointestinal disorders and it is commonly used in farm animals [17, 18].

Previous studies have suggested the transabdominal ultrasound approach of the rumen as a possible diagnostic tool for subacute rumen acidosis in dairy cattle. The wall of the rumen could be identified as a thick echogenic line (3.0–4.8 mm) adjacent to the left abdominal wall from the left flank of dairy cows [19]. Mirmazhari- Anwar et al. (2013) [20] reported increasing rumen mucosa thickness when using a transabdominal rumen ultrasound in cannulated bulls fed diets, increasing in concentrate level from 5 to 96% dry matter (DM). Neubauer et al. (2018) [21] confirmed the potential of rumen mucosa thickness measurement via ultrasound as a diagnostic tool for subacute rumen acidosis, adjusted for the age or lactation number of the individual dairy cow.

Therefore, the aim of this study was to evaluate the potential suitability of the transabdominal ultrasonographic examination of the ruminal wall to diagnose of chronic rumen acidosis in beef cattle, as a fast a non-invasive tool to be used in field condition, compared to direct measurement of ruminal pH.

## Results

Data showed after 15 ± 5 days of the housing that two hundred eighty-nine fattening bulls (60.5%) had a rumen pH > 5.9, while one hundred eighty-nine (39.5%) had a rumen pH < 5.9 and respectively one hundred forty-eight (30.9%) in chronic rumen acidosis (5.5 < pH ≥ 5.8) and forty-one (8.5%) in the risk of acute rumen acidosis (pH < 5.5) [9, 16].

Table 1 showed the values regarding the descriptive analysis of the results found for the rumen pH, RWT, RMST and VFA (acetic acid, propionic acid, butyric acid).

**Table 1:** Descriptive data (mean, standard deviation, minimum and maximum value, median, 5th, and 95th percentile) regarding the rumen pH, the ultrasonographic measurements of RWT and RMST and VFA (acetic acid, propionic acid, butyric acid)

	Total Population (*n* = 478)					
					Percentiles	
Parameters	Mean	SD	Minimum-Maximum value	Median	5th	95th
Rumen pH	6	0.4	4.6–7.2	6	5.7	6.3
Rumen wall thickness (mm)	8.1	2.2	4.7–16.1	7.7	6.3	9.5
Rumen mucosa and submucosa thickness (mm)	5.7	1.8	2.9–11.4	5	4.2	7
Acetic Acid (mg/ml)	3.1	0.5	0.9–4.4	3.1	2.7	3.5
Propionic acid (mg/ml)	1.3	0.3	0.3–2.5	1.2	1.1	1.5
Butyric Acid (mg/ml)	0.9	0.2	0.1–1.7	0. 9	0.8	1.1

The Analysis of Variance (ANOVA) model showed that there was the high significant effect of the pH class for RWT and RMST (P < 0.001) and the equate significant effect of rumen muscularis and serosa (*P* = 0.014). For RWT and RMST poc-hoc pairwise comparisons among levels showed that normal class was statistically different from the other two (chronic rumen acidosis and risk of acute rumen acidosis). Based on these results, animals were grouped as healthy (normal class) and chronic rumen acidosis (chronic rumen acidosis and risk of acute rumen acidosis class).

Spearman RANK correlation analysis showed interaction between rumen pH and RWT (− 0.71; *P* < 0.0001) and RMST (− 0.75; P < 0.0001). A significant Spearman’s correlations (Table 2) were found between VFA, RWT and RMST.

**Table 2:** Spearman’s Correlation (r values) and statistical significance (*P* values) between parameters investigated

Parameters	r	P
Rumen wall thickness		
Rumen pH	−0.71	< 0.0001
Acetic acid	0.37	< 0.0001
Propionic acid	0.46	< 0.0001
Butyric acid	0.39	< 0.0001
Rumen mucosa and submucosa thickness	0.94	< 0.0001
Rumen mucosa and submucosa thickness		
Rumen pH	−0.75	< 0.0001
Acetic acid	0.30	0.005
Propionic acid	0.47	< 0.0001
Butyric acid	0.34	< 0.0001
Rumen pH		
Acetic acid	−0.50	< 0.0001
Propionic acid	−0.66	0.001
Butyric acid	−0.44	< 0.0001

**Fig. 1:**
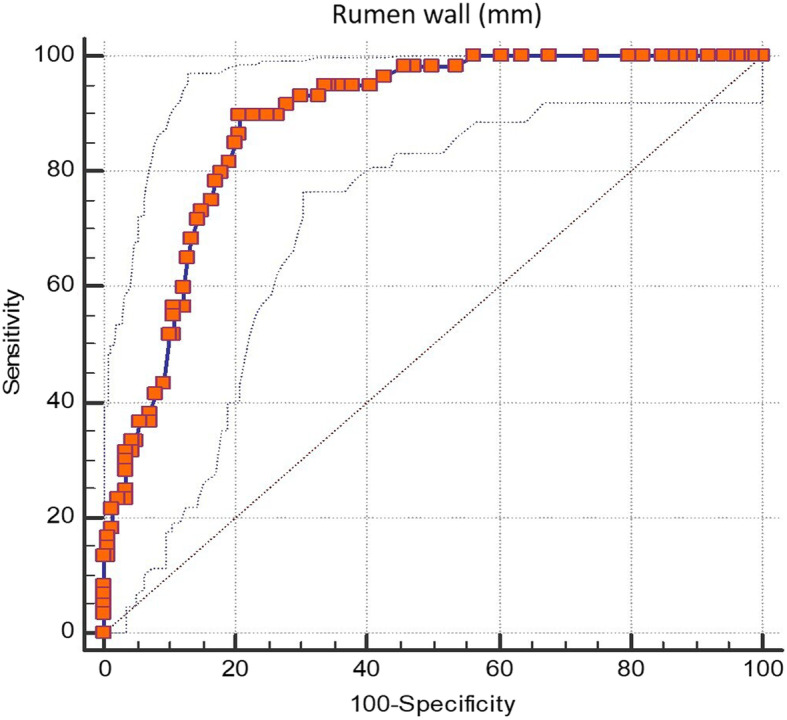
Receiver operating characteristic (ROC) curve analysis of the rumen wall thickness for detecting chronic acidosis. The best cut-off value is > 8.2 mm, (area under the ROC curve of 0.88: *P* < 0.0001; 95% c.i. 0.83 to 0.98; sensitivity 90%; specificity 79%; positive likelihood ratio 4.34; negative likelihood ratio 0.13). criterion: Youden index

The differentiation efficiency of RWT between healthy and chronic rumen acidosis groups, as a result of the receiver operator curve (ROC) analysis, was quite good with an area under the receiver operator curve (AUROC) of 0.88: *P* < 0.0001; 95% CI: 0.83–0.98. Using a cut-off value of > 8.2 mm, calculated by Youden index, sensitivity was 90% and specificity was 79%, positive likelihood ratio = 4.34, negative likelihood ratio = 0.13 (Fig. 1).

**Fig. 2:**
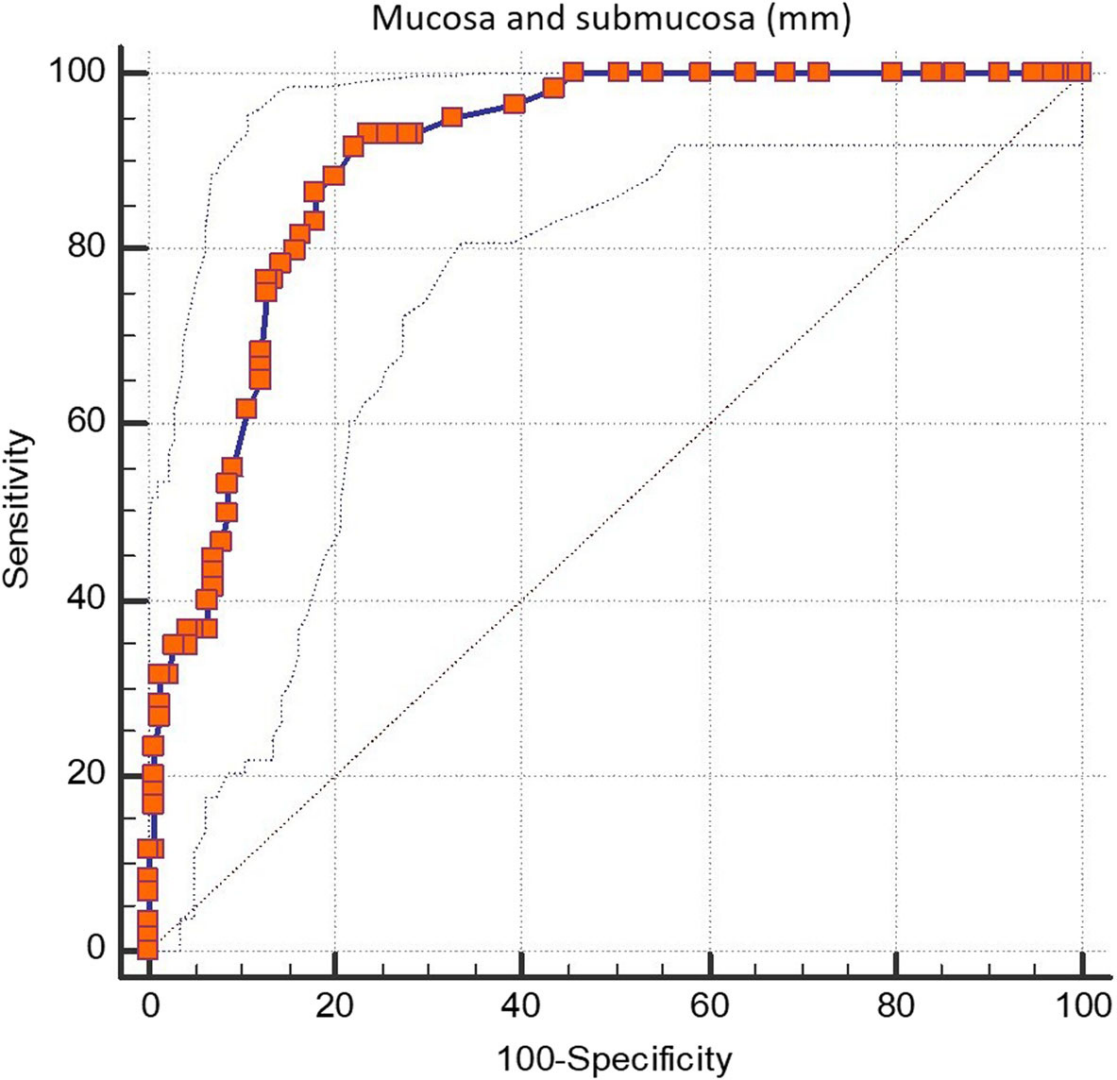
Receiver operating characteristic (ROC) curve analysis of the mucosa and submucosa thickness for detecting chronic acidosis. The best cut-off value is > 5.3 mm, (area under the ROC curve of 0.90: P < 0.0001; 95% c.i. 0.85 to 0.94; sensitivity 93%; specificity 76%; positive likelihood ratio 3.96; negative likelihood ratio 0.08). criterion: Youden index

The differentiation efficiency of RMST between healthy and chronic rumen acidosis groups, as a result of ROC curve analysis, was good with an AUROC of 0.90: *p* < 0.0001; 95% CI: 0.85–0.94. Using a cut-off value of > 5.3 mm, calculated by Youden index, sensitivity was 93% and specificity was 76%, positive likelihood ratio = 3.96, negative likelihood ratio = 0.08 (Fig. 2).

## Discussion

The results of our study show that transabdominal ultrasound measurements of the rumen wall thickness represent a useful tool and a potentially suitable method to diagnose chronic rumen acidosis in beef cattle. Ruminal acidosis continues to be a common ruminal digestive disorder in beef cattle and can lead to marked reductions in cattle performance [9]. Our first result showed the effect of the increase dietary concentrate during the receiving period in fattening cycle. The 39.5% of all animals included in the study had a rumen pH lower than the threshold value of 5.8 after 15 ± 5 days of the housing.

The decrement of the pH value may be attributed to the sudden change of the diet and to the consequential lack of adaptive capability of the bulls. Diets with high-concentrate content leading to a moderate decline in ruminal fluid pH were consistently reported to trigger an increase in length of ruminal papillae, dilatation and hyperemia of mucosal and submucosal capillaries as well as submucosal edema, thereby causing a marked increase in thickness of ruminal mucosa. Therefore, the thickening of the rumen mucosa was well correlated with the decrement of rumen pH [7, 22].

In our study, the higher correlation between ruminal pH and RMST could largely explain this (r = 0.75, *P* < 0.0001). According to Mirmazhari-Anwar et al. (2013) [20] found also a higher correlation between ruminal pH and rumen mucosa thickness (r = 0.818, *P* < 0.01) in adult dairy bulls fed diets varying in concentrate levels from 5% up to 96%, which consequently led to a more extreme range in ruminal pH (5.2–7.1). Moreover, in this study RWT and RMST had a significant correlation to VFA determinate: acetic acid, propionic acid, butyric acid. This is explained by increased concentrations of VFA in the rumen that are primarily stimulants for papillary development [23, 24]. The VFA were accepted to be the main trigger for growth of rumen papillae wherever the mucosa was directly exposed to these acids [23, 24]. The adaptation of morphological dimensions of ruminal epithelia could be functionally fast and strong, because the epithelia has to cope with the rapid increase in VFA load to which they are exposed [25, 26]. Furthermore, recent studies suggested that an increase in ruminal VFA and dietary particle sizes can alter expression of genes (like Insulin-like growth factor-binding proteins) involved in cell proliferation/apoptosis process involved in the adaptation of the rumen epithelium [25, 27]. The higher production of VFA, particularly butyrate and propionate, and a higher absorption from the rumen by the mucosa, in a low ruminal pH, will lead to a parakeratosis of the ruminal epithelium that consequently can cause a rumenitis. Rumenitis is a frequent sequel to rumen acidosis [25, 28].

Mirmazhari- Anwar et al. (2013) [20] determined a cut-off value of 7.3 mm for the rumen mucosa thickness to identify animals with ruminal fluid pH < 5.5 approximately 4 h post feeding. This cut-off value was obtained to measurements of rumen mucosa thickness by 8 MHz linear transducer in five cannulated bulls fed diets, increasing in concentrate level (5–96% DM).

Our study included four hundred seventy-eight beef cattle and cut-offs of > 5.4 mm and > 8.2 mm for RMST and RWT, respectively, were determined for distinguishing animals with ruminal pH ≤ 5.8.

However, this study has methodological limitations. First, the research was limited because we used a retrospective study design, adherence to a coherent standardized protocol to get repeatability of ultrasound measurements was not possible, although the high number of the ultrasound evaluation performed by a single observer with the same portable ultrasound scanner. Second, the limited axial resolution ~ 0.3 mm of a 6.6 MHz probe was used in the study that limits the smallest detectable difference in thickness. Thus, evaluation of RWT and RMST with higher frequency probes that have higher axial resolution with more accurate measurement it could lead to more reliable results.

Further studies will be necessary to evaluate whether RWT and RMST could reach a plateau at a certain time or a ratio of concentrate feeding and to what extent diminished cell function and parakeratosis occurs as hypothesized by Neubauer et al. (2018) [21].

Although the sample of the animals in the study was considerable, we had investigated just beef cattle of the same sex, breed and stage of breeding, other several variables should be considered seeing if there are differences in RWT and RMST measurements like in dairy cows [21].

## Conclusions

Diagnostic ultrasound is a useful tool if employed correctly, enabling non-invasive and potentially rapid evaluation of areas of interest. Transabdominal ultrasonography of the RWT and RMST has established its potential to be a suitable diagnostic tool, fast, non-invasive and easily applicable to be used in field condition to identify beef cattle affected by chronic rumen acidosis.

## Methods

This retrospective study was carried out from historical clinical records of the Ruminant Clinic of the Padua University Veterinary Teaching Hospital and as part of doctoral studies.

We performed this retrospective study from a dataset recorded from the extramural clinical activity of the Preventive Medicine Service and Breeding of Ruminants Clinic (Veterinary Teaching Hospital, University of Padua - OVUD) from 2013 to 2017. The reasons for a retrospective study rather than prospective, is a lack of existing literature on the topic of beef cattle, low expensive methods and simplicity.

From a dataset of 1389 animals (37 beef cattle farms) assessed by rumenocentesis and ruminal wall ultrasound on the clinical examination, 478 beef cattle were selected based on the following inclusion criteria: sex, breed and stage of breeding.

The animals were young fattening bulls of Charolaise breed, imported from France with an average body weight of 424.5 ± 33.4 kg and an average age of 10.8 ± 0.7 months at 15 ± 5 days after housing. The arriving cattle were fed a high long fiber acclimation diet for 5–7 days before moving on to the growth diet. After that period, all animals were fed with a finishing diet mainly based on maize (silage and high moisture ear), soya bean meal, dry sugar beets and wheat straw. The diet was provided daily as a total mixed ration (TMR) for ad libitum intake based on 10% feed refusal (as-fed basis). Dry matter intake (DMI) mean values were recorded for all beef cattle farms (mean values: DMI: 18.0 ± 1. 5 Kg per animal; DM: 9.78 ± 0. 8 per animal). Drinking water was available ad libitum, through two bowls per pen. All animals were housed in boxes of 10 in individual concrete-floor tie stalls within an enclosed barn.

### Ultrasonography, rumenocentesis and evaluation of rumen fluid pH

The ultrasound and rumenocentesis evaluations were performed at 15 ± 5 days after housing. Animals were secured in standing position in a crate.

The transtransabdominal ultrasound of the ruminal wall was performed with a portable ultrasound scanner (MyLabOne™, Esaote S.p.a.), equipped with a multi-frequency convex probe (2.2–4.3-6.6 MHz; SC3421, Esaote S.p.a.). All ultrasonographic examinations were performed by the same operator.

Ultrasonographic images were taken on the left side, from the ventral sac of the rumen, approximately 15–20 cm caudal and ventral to the costocondral junction of the last rib, the same rumenocentesis area [16]. Ultrasonographic examination of the rumen wall was conducted 5 min before the rumenocentesis. The examination window was clipped and cleaned with alcohol, and lubrication gel was applied to ensure the examination position and proper conduction.

Ultrasonographic settings were maintained constant throughout the all scans: frequency 6.6 MHz, depth 8.0 cm, gain 90.0%, time gain compensation was in a neutral position.

Ultrasound image was taken when the ruminal wall rested against the abdominal wall in a relaxed state at the end of the ruminal contraction and the probe marker was orientated towards the patient’s head.

**Fig. 3:**
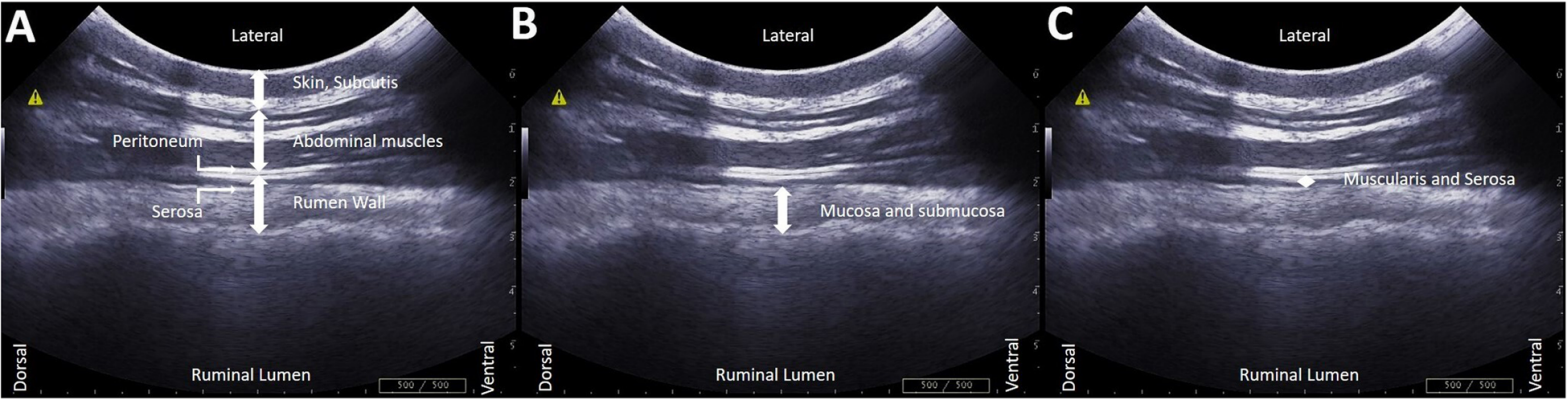
Ultrasonographic image of the abdominal and rumen wall from the left flank showing (**a**) from top to bottom skin and subcutis, abdominal muscles, peritoneum, serosa and rumen wall (**b**) mucosa and submucosa of the rumen (**c**) muscolaris and serosa of the rumen

Measurements were obtained from a single image of the rumen. RWT and RMST were measured using the distance measurement function of the software MyLab™Desk (Esaote) (Fig. 3). The rumen wall has a characteristic appearance of three distinct sonographic layers: starting at the inner surface of the rumen the first hyperechoic layer corresponds to the mucosa and submucosa (the two layers cannot be distinguished one from the other), the second hypoechoic layer is the muscularis and the third hyperechoic layer corresponds to the serosa [20, 21].

A sample of rumen fluid was taken from each animal by the rumenocentesis method as described by Nordlund and Garrett (1994) [11] and modified by Gianesella et al. (2010) [16]. Samples of rumen fluid were collected between 4 and 6 h after TMR administration because during this interval the rumen pH reaches the peak of acidity [6, 29]. From each animal was collected 15 ml of rumen fluid and its pH was determined using a digital portable pH meter (Zetalab PC70, XS-instruments, Padova, Italy).

The VFA were determined using an aliquot of 8 ml of ruminal liquid which was immediately acidified with 2 mL of hydrogen chloride (HCl 0.6 M) and stored at 4 °C until the samples arrived at the laboratory where they were stored at − 20 °C until analysis.

The VFA contents were measured on the supernatant of the rumen fluid samples obtained by centrifugation (1300 × gfor 15 min) using an HPLC Perkin Elmer Series 10, mobile phase H2SO40.0025 N, flux 0.6 mL/min, detector Waters 410, column Gecko 2000 at a working temperature of 60 °C [5]. VFA were determined: acetic acid, propionic acid, butyric acid.

### Statistical analysis

All statistical analyses were performed using SAS (SAS 9.3, Institute Inc., Cary, NC).

Ultrasound measurements were submitted to One-Way Analysis of Variance (ANOVA) (PROC GLM) considering the effect of the pH class (normal vs chronic rumen acidosis vs risk of acute rumen acidosis: normal > 5.9; chronic rumen acidosis between 5.5–5.8; risk of acute rumen acidosis < 5.5). All pairwise contrast between least squares means were performed using the Bonferroni correction. Normality of residuals was evaluated by Shapiro-Wilk test (PROC UNIVARIATE) and values ≥0.9 were considered as normal. Based on the results of ANOVA, animals were grouped as healthy and chronic rumen acidosis.

Spearman RANK correlation analysis between ultrasound measurements and the rumen fluid parameters were calculated.

ROC curve analysis (MedCalc) was performed to calculate on ultrasound measurements the threshold that discriminates healthy vs chronic rumen acidosis groups.

The threshold was identified using the Youden criterion. The area under the ROC curve (AUROC) as taken as an index of accuracy.

## Data Availability

The datasets used and/or analysed during the current study are available from the corresponding author on reasonable request.

## Abbreviations

AUROC: Area under the receiver operator curve
DM: Dry matter
DMI: Dry matter intake
RMST: Rumen mucosa and submucosa thickness
ROC: Receiver operating characteristic
RWT: Rumen wall thickness
SD: Standard deviation
TMR: Total mixed ration
VFA: Volatile fatty acids
